# A runtime alterable epidemic model with genetic drift, waning immunity and vaccinations

**DOI:** 10.1098/rsif.2021.0648

**Published:** 2021-11-24

**Authors:** Wayne M. Getz, Richard Salter, Ludovica Luisa Vissat, James S. Koopman, Carl P. Simon

**Affiliations:** ^1^ Department ESPM, UC Berkeley, Berkeley, CA 94720-3114, USA; ^2^ School of Mathematical Sciences, University of KwaZulu-Natal, Durban, South Africa; ^3^ Numerus, 850 Iron Point Rd., Folsom, CA 95630, USA; ^4^ Computer Science Department, Oberlin College, Oberlin, OH 44074, USA; ^5^ School of Public Health, University of Michigan, Ann Arbor, MI 48109, USA; ^6^ Center for the Study of Complex Systems, University of Michigan, Ann Arbor, MI 48109, USA; ^7^ Gerald R. Ford School of Public Policy, University of Michigan, Ann Arbor, MI 48109, USA; ^8^ Department of Mathematics, University of Michigan, Ann Arbor, MI 48109, USA

**Keywords:** SEIR individual-based models, escape mutations, RAMPs, SARS-CoV-2, pathogen variants, vaccination

## Abstract

We present methods for building a Java Runtime-Alterable-Model Platform (RAMP) of complex dynamical systems. We illustrate our methods by building a multivariant SEIR (epidemic) RAMP. Underlying our RAMP is an individual-based model that includes adaptive contact rates, pathogen genetic drift, waning and cross-immunity. Besides allowing parameter values, process descriptions and scriptable runtime drivers to be easily modified during simulations, our RAMP can used within R-Studio and other computational platforms. Process descriptions that can be runtime altered within our SEIR RAMP include pathogen variant-dependent host shedding, environmental persistence, host transmission and within-host pathogen mutation and replication. They also include adaptive social distancing and adaptive application of vaccination rates and variant-valency of vaccines. We present simulation results using parameter values and process descriptions relevant to the current COVID-19 pandemic. Our results suggest that if waning immunity outpaces vaccination rates, then vaccination rollouts may fail to contain the most transmissible variants, particularly if vaccine valencies are not adapted to deal with escape mutations. Our SEIR RAMP is designed for easy use by others. More generally, our RAMP concept facilitates construction of highly flexible complex systems models of all types, which can then be easily shared as stand-alone application programs.

## 1. Introduction

Kermack and McKendrick pioneered the application of differential equations to modelling the dynamics of disease systems that included susceptible (*S*), infected/infectious (*E*/*I*) and recovered (*R*; we use *V* to include vaccinated) classes of individuals [1]. Subsequent extensions of their formulation include, *inter alia*, additional disease and demographic classes [2], multihost and pathogen strain considerations [3,4], spatial heterogeneity [5,6], network [7] and individual-based formulations [8,9]. Along with these extensions has come the challenge of ‘not being able to see the forest for the trees’ when questions beyond those pertaining to the profiles of epidemics on homogeneous, well-mixed, large populations arise. As with the current COVID-19 pandemic, these questions may relate to the emergence of new pathogen variants [10], the effects of waning and cross-immunity in hosts with different exposure histories to these variants [11], differential transmission and virulence of these variants, issues of spatial heterogeneity and host heterogeneity related to age, gender, and health status factors [12].

From the points of view of both technology and human comprehension, we only have the capacity to understand how a limited number of factors may explain or affect epidemiological outcomes at any one time when measures are applied to mitigate the severity of disease outbreaks. Thus, we are brought to consider the issue of how to craft a model so that it has the appropriate level of complexity to address the questions at hand [13,14]. We otherwise follow Einstein’s dictum that ‘models should be as simple as possible, but no simpler.’

To facilitate the processes of both incorporating complexity into and stripping complexity out of models, we have developed the concept of a Runtime Alterable Model Platform (RAMP). This allows us to focus on outcomes rather than on the logistics of modifying and coding models and carrying out comparative analyses. Our RAMP includes panels, windows and sliders that allow users to specify and manipulate model parameter values and to modify process function descriptions, and scripting drivers for implementing sets of simulations. Furthermore, modifications can be made both at the start of and during the course of a simulation, while protecting the integrity of the underlying code. In addition, our RAMP automates documentation of all parameter values, process descriptions, changes and actions (modifications and substitutions during simulation) in a file that is then saved at the end of each simulation. This file is then ready for later comparative analyses across sets of simulations, or within a data processing environment that incorporates our RAMP as a component package, such as R-Studio.

RAMPs can be developed for models that address classes of problems, formulated using a Goldilocks principle. Thus, these classes should not be too general so that comparisons within each class require extensive alterations to models (members of the class should share significant structural properties with regard to process dynamics) but also not too specialized so that comparisons across members of the class are not too limited to provide answers to questions of interest. Thus we might develop different RAMPs to study genetic, morphogentics, epidemiological, evolutionary, geological and environmental processes.

**Figure 1.**
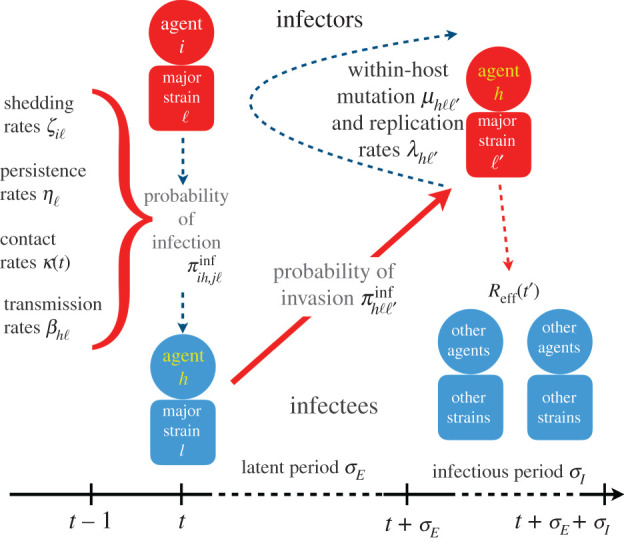
An overview of the processes included in our M-SEIR model (see table 1 for equation references). The probability $\pi_{ih,jl}^{\inf}$ of *A*_*h*_ being infected primarily with pathogen ℓ in terms of receiving an effective dose from agent *A*_*i*_ is computed in terms of a concatenation of shedding rates (*ζ*_*i*ℓ_), environmental persistence rates (*η*_ℓ_) and host transmission (*β*_*h*ℓ_) processes (electronic supplementary material, equation (A.12)) and includes both waning and cross-immunity factors. The probability $\pi_{h\ell \ell^{\prime }}^{\mathrm{inv}}$ that the dominant variant emerging in host *A*_*h*_ is variant ℓ′ given initial infection with variant ℓ is computed in terms of within-host mutation and within-host replication process (electronic supplementary material, equation (A.13)) and also includes both waning and cross-immunity factors. These two probabilities are then used to compute the overall probability *π*_*ih*,*j*ℓ′_ (electronic supplementary material, equation (A.14)) that infector *i*, infected with major variant *j*, infects infectee *h* with major variant ℓ′. The quantity *R*_eff_(*t*′) is the expected number of individuals each infectious agent is expected to infect around time *t*′ ∈ [*t* + *σ*_*E*_, *t* + *σ*_*E*_ + *σ*_*I*_], where *R*_0_ = *R*_eff_(0) is estimated for our model using electronic supplementary material, equation (A.26).

Here, we provide an example of a RAMP that has sufficient breadth to investigate an array of questions pertaining to multivariant epidemiological dynamics for directly transmissible diseases, such as the current SARS-CoV-2 pandemic [15,16], influenza [17] or Ebola [13,18]. For simplicity, we refer to this as our M-SEIR (multivariant susceptible-exposed-infected-recovered) RAMP. For the study of water-borne or vector-borne diseases, similar but somewhat more complicated RAMPs will need to be developed. Our M-SEIR RAMP is designed to be used by individuals either with no coding skills, or with minimal coding skills if they desire to modify some of the process descriptions incorporated into the supplied platform. It is sufficiently detailed, however, to allow the user to incorporate either supplied or user-altered versions of the following processes: (i) pathogen variant-specific shedding [19], environmental persistence [20], within-host replication [21] and mortality rates [22]; (ii) immunological waning with variant cross-immunity [23,24]; (iii) pathogen variant drift during transmission and within-host replication [25]; (iv) an adaptive contact rate [26]; (v) a time-dependent, uni- or multivalent vaccine rollout [27,28] (figure 1; for mathematical details, see §2, and electronic supplementary material, appendix A).

**Figure 2.**
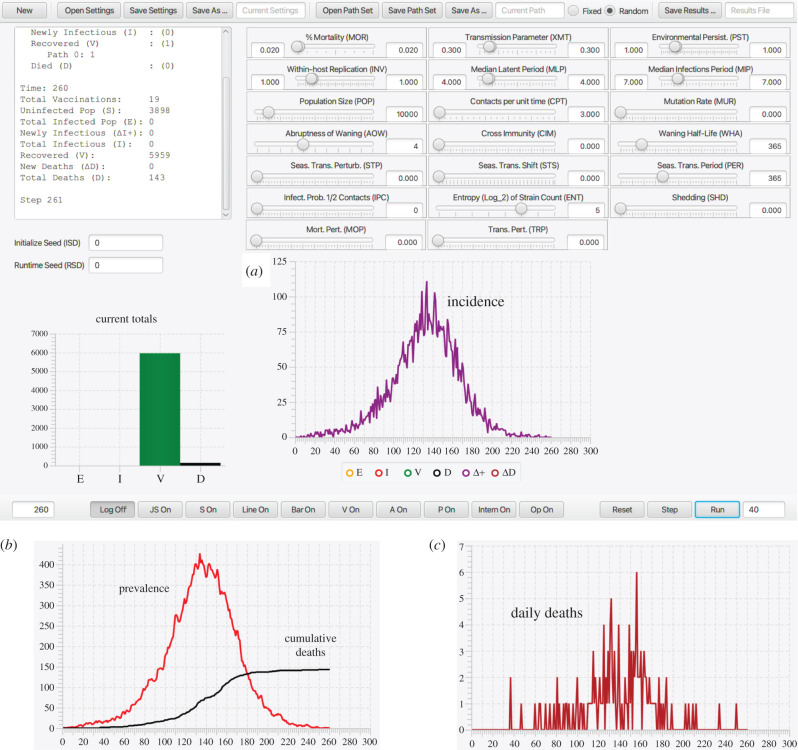
(*a*) The dashboard of our Java Runtime-Alterable-Model Platform (RAMP) SEIVD (*S* = susceptibles, *E* = exposed, *I* = infectious, *V* = immune, *D* = dead) individual-based model (IBM) and simulations obtained using the parameters values depicted in the slider windows (also see table 2). The top left window of this dashboard contains information on the final state of the population (in this case *S* = 3898 and *D* = 143 in a population of *N*_0_ = 10 000), the bottom left bar graph of the dashboard panel gives the final values of *E*, *I*, *V* and *D* at epidemic cessation at time *t* = 166 (days) or the simulation run time, whichever comes first. The dashboard also shows a graph of incidence (purple: selected using coloured buttons below the graph). The bottom ribbon of the dashboard has a series of radio buttons that respectively open a log, a JavaScript (JS) and a scripting (*S*) window, line and bar graph windows (for multivariant runs), as well as windows for controlling vaccination strategies (*V*), listing realtime agent information (*A*), pathogen parameter values (*P*), monitoring probability computations (Intern), coding and controlling runtime alternative operations (Op), and three runtime buttons (Reset, Step, Run). (*b*) Graphs of prevalence and cumulative deaths (cut out from main panel when only the red and black buttons are on) and (*c*) daily deaths (crimson button) are pasted below the dashboard.

The reason for our inclusion of an adaptive contact rate process is that the local nature of contact rate patterns is well established as an important driver of outbreak dynamics [15]. If contact rates remain unchanged during the course of an epidemic, then a classic incidence curve (as in figure 2) will be the result. However, repeated peaks associated with consecutive outbreak waves arise as a result of implementing and then relaxing social distancing measures [15]. In the absence of social distancing drivers, which vary greatly from one location/region/country to another, an automated way to evaluate the effects of social distancing measures is through an adaptive contact process of the type that we include in our M-SEIR RAMP.

To illustrate the application of our M-SEIR RAMP, we used it to explore aspects of disease incidence and prevalence profiles using parameters that are applicable to the SARS-CoV-2 pathogen at the start of the COVID-19 pandemic. For example, we compare constant and adaptive (viz., prevalence-dependent) contact rate processes under different waning immunity scenarios. We also explore the emergence of variants for different mutation and variant transmission rates. Additionally, we show how our M-SEIR RAMP can be used to evaluate the efficacy of uni- and multivalent vaccines applied at various time-dependent rates, where choice of valency may switch in response to realtime monitoring and surveillance data. Such adaptive vaccination programs may be required to combat the evolutionary arms race between vaccine efficacy and the evolution of new pathogen variants [25,28,29]. We hope, however, that our results and subsequent investigations using our M-SEIR RAMP will provide the kinds of quantitative analyses that can help formulate highly effective local- or country-level vaccination programs that avoid some of the vaccination rollout pitfalls revealed by our analysis, as well as encourage the adoption of effective adaptive vaccination programs.

## 2. Material and methods

### 2.1. Our M-SEIR in a nutshell

We constructed an individual-based model (IBM) of a susceptible-exposed-infectious-recovered (i.e. an SEIVD model, where removed *R* are split into *V* = immune/vaccinated, and *D* = dead) epidemiological process [30,31] in a homogeneous population with a random encounter contact rate parameter *κ*_0_ > 0. Our formulation allows for the emergence of multiple variants of the pathogen during a sequence (i.e. concatenation) of process depicted in figure 1 and listed in table 1. Specifically, our formulation includes a host immunological waning process [23,32] and a mutational process that impacts both transmission of mutant variants from the infectee and genetic drift [11,24,33] of variants within the infector, with rates impacted by cross-immunity effects. We also allowed for variation in pathogen variant transmissibility (i.e. in the *β* > 0 parameter of the frequency dependent transmission function *βSI*/*N* [34,35]) and pathogen virulence as represented by the disease-induced host mortality rate in the sense of Anderson & May [36] (and often represented by a parameter *α* ≥ 0 [34]).

The detailed formulation of our model and its algorithmic implementation is provided in appendix A (electronic supplementary material, SOF), with references to relevant equations in this provided in table 1. In a nutshell we:

1. Defined a set of 2^*J*^ pathogen variants (user selected value for variant entropy *J* ranging from 0 to 7; pathogen index *j* = 0, …, 2^*J*^ − 1) with a genetic-relatedness topology of a *J*-dimensional unit cube—i.e. each pathogen has *J*-loci that can take on one of two allelic values at each locus with immediate neighbouring variants differing from each other by exactly one allelic value (0 or 1) at only one of the *J* loci.
2. Defined a population of *N*_0_ hosts as belonging at time *t* to either an epidemiologically naive set of susceptible individuals **S** of size *N*_*S*_(*t*), a set **A** of *N*_*A*_(*t*) identified agents *A*_*i*_ (*i* = 1, …, *N*_*A*_(*t*)) whose epidemiological histories are known, or a set **D** of *N*_*D*_(*t*) individuals that have died from the disease.
3. Allowed pathogen variant-specific transmission ‘force’ (${\bar{\beta }}_{\;j}>0$) and virulence (*α*_*j*_ ≥ 0) parameters to vary in value among one another within a defined range ${\bar{\beta }}_{j}\in [\beta_{\min},\beta_{\max}]$ and *α*_*j*_ ∈ [*α*_min_, *α*_max_].
4. Kept track of the total prevalence *N*_*I*_ as the sum of the prevalences of the individual variants $N_{I_{j}}$, *j* = 0, …, 2^*J*^ − 1—i.e. $N_{I}=\sum_{\;j=0}^{2^{J}-1}N_{I_{j}}$.
5. Introduced a random contact rate function *κ*(*t*) with a constant parameter *κ*_0_ that is Poisson distributed on [*t*, *t* + 1), *t* = 0, 1, …, multiplied by an adaptive response function that reduces the contact rate with increasing disease prevalence, such that the *κ*(*t*) is reduced to *κ*_0_/2 when the $N_{I}(t)/(N_{0}-N_{D})=p_{I}^{\mathrm{half}}$—see electronic supplementary material, equation (A 7)
6. Updated the epidemiological state of the agents *A*_*i*_ with respect to each of the variants *j* = 0, …, 2^*J*^ − 1, where the state with respect to particular variant *j* at time *t* is represented by a list that includes the following *J* entries pertaining to the state of *A*_*i*_ with respect to pathogen variant *j* = 0, …, 2^*J*^ − 1. If the *j*th entry is:
  (a) 0, then agent *A*_*i*_ has never been infected with this variant
  (b) *E*_*j*_(*t*, *τ*_*ij*_), then agent *A*_*i*_ was infected at time *τ*_*ij*_ ≤ *t* with this variant, but is not yet infectious for an expected period of *σ*_*E*_ units of time
  (c) *I*_*j*_(*t*, *τ*_*ij*_), then agent *A*_*i*_ was infectious at time *t* with this variant, for an expected period of *σ*_*I*_ units of time, having been most recently infected (reinfections with the same variant may occur) with this variant at time *τ*_*ij*_ < *t*
  (d) *V*_*j*_(*t*, *τ*_*ij*_), then agent *A*_*i*_ has now recovered from its most recent infection at time *τ*_*ij*_ with this variant and has some level of waning immunity to it

7. Assumed that agent *A*_*i*_ can be infectious at time *t* with at most one dominant variant (denoted by the index *j*), although due to mutational processes this agent may infect other agents with variants related to this dominant variant at much lower rates (i.e.through application of a mutation factor *μ* ≪ 1, applied in our basic model through electronic supplementary material, equation (A.13)).
8. Assumed that agent *A*_*i*_ will have different levels of waning immunity to all of the variants to which it has been infected in the past.
9. Included waning immunity functions *ω*_*ij*_(*t*) (electronic supplementary material, equation (A 6)) used to compute the level of immunity that agent *A*_*i*_ has to its most recent infection by variant *j*.
10. Included cross-immunity effects (a *J*^2^-matrix *C*) that apply both to the *infector* transmitting the pathogen and the *infectee* being invaded (inv; its ‘airport code’ as described in electronic supplementary material, figure B.3) by the pathogen of interest, both of which reduce the likelihood of infection and variant drift by variant *j* compared with closely related variants ℓ (for a simple example of the matrix *C*, see equation (2.1) in §3 below).
11. Computed an *infection probability* $\pi_{ih,j\ell }^{\inf}$ that agent *A*_*i*_ infected with variant *j* infects agent *A*_*h*_ with variant ℓ in terms of a concatenation of infector viral shedding (*ζ*_*i*ℓ_; for a simple example see equation (2.6) in §3 below), viral persistence in the environment (*η*_ℓ_), and viral transmission (*β*_*h*ℓ_) processes (figure 1)
12. Computed an *invasion probability* $\pi_{h\ell \ell^{\prime }}^{\mathrm{inv}}$ that an agent *A*_*h*_ infected with variant ℓ becomes infectious with variant ℓ′ as its major variant, in terms of the multiplicative effects of viral mutation (*μ*) and replication (*λ*_ℓ_) processes ongoing within an infectee *A*_*h*_ during this infectees exposed ($E_{\ell^{\prime }\tau_{h\ell^{\prime }}}$) and infectious ($I_{\ell^{\prime }\tau_{h\ell^{\prime }}}$) periods (figure 1).
13. Computed the overall probability *π*_*ih*,*j*ℓ′_ that an infector *A*_*i*_ infected with major variant *j* results in an infectee *A*_*h*_ expressing ℓ′ as its major variant.
14. Assumed that waning and cross-immunity to a particular variant is the same for both natural infections and vaccinations that use the antigen associated with that variant (of course we can easily extend our model to remove this assumption once data become available to support different waning and cross-immunity rates for natural infections and particular vaccines).
15. Implemented a discrete time individual-based stochastic SEIVD (here *V* represents individuals that have either recovered from infection or have been vaccinated, *D* represents cumulative dead; also see [37]) multivariant model that includes specifiable time-dependent univalent and multivalent vaccination implementations.

**Table 1.** Variables, indices and functions in our M-SEIR RAMP.

symbols	variables and indices	equation
		(see electronic supplementary material)
*variables*		
*t*	time (variable and function values depend on time)	
*N*_*S*_, *N*_*A*_, *N*_*D*_	size of sets **S**, **A** and **D**	equation (A.1)
*J*, *j*, *m*, ℓ and ℓ′	variant entropy and indices (0, …, 2^*J*^ − 1)	equation (A.2)
*N*_*I*_, $N_{I_{j}}$	total and variant *j* infectious class size	
*A*_*i*_, *A*_*h*_	specific agents *i*, *h* = 1, …, *N*_*A*_(*t*) in set **A**	equation (A.3)
***functions*** (if a parameter now it may be elaborated later as a function)		
*κ*	adaptive contact rate	equation (A.7)
*ω*_*ij*_	waning immunity of *A*_*i*_ w.r.t. variant *j*	equation (A.6)
*c*_*mj*_	cross-immunity encountered by variant *j* when agent previously infected with variant *m*	equation (A.8)
*ϕ*_*ij*_	immunity modifier	equation (A.8)
*ζ*_*ij*_	shedding rate of variant *j* by infector *A*_*i*_	equation (A.9)
*η*_ℓ_	environmental persistence	equation (A.10)
*β*_*h*ℓ_	variant transmission to infectee *A*_*h*_	equation (A.11)
$\pi_{ih,j\ell }^{\inf}$	probability *A*_*i*_ infects *A*_*h*_	equation (A.12)
*μ*	mutation process factor	equation (A.13)
*λ*_ℓ′_	within-host replication rate	equation (A.13)
$\pi_{h\ell \ell^{\prime }}^{\mathrm{inv}}$	probability ℓ′ is major variant when ℓ invades	equation (A.13)
*π*_*ih*,*j*ℓ′_	probability ℓ′ is major variant in *A*_*h*_when *j* is major variant in *A*_*i*_	equation (A.14)

### 2.2. Our RAMP implementation

Models of systems process can be coded: (i) directly using highly efficiently compilable computer languages (e.g. C++, FORTRAN, Java); or (ii) less efficiently, but more easily, using scriptable (e.g. JavaScript, Python, Perl) computer languages. More conveniently and expeditiously, they can be coded up, as discussed in [38], using a systems modelling platform that contains precoded modules and graphical elements, such as Matlab’s SIMULINK, Mathematica, Stella, AnyLogic, Numerus or Berkeley Madonna. Advantages of the latter include more rapid and accurate model development, though simulations may be slowed down by platform overhead. Between these extremes, we propose a more general approach to specific classes of systems’ models, where the basic system structure is fixed, but implementation of some elements can be easily and safely altered so that optional implementations are presented at runtime. We call such a design *runtime alterable-model platforms*. (RAMPs); and here we present a Java RAMP implementation of the M-SEIR described in the previous subsection.

The characteristics we envision for a model platform to be a RAMP are:

1. RAMPs include a set of model parameters (constants) whose values can be selected or specified (sometimes within a predefined range of values) at simulation runtime using a switch, nob, slider or text-entry window accessed via a platform graphical interface or dashboard (see figure 2 and table 1 which apply to our M-SEIR RAMP).
2. RAMPs include a specific set of *runtime alternative modules*, (RAMs), where the original can be redefined in a graphical interface window, and the unaltered original and the alternative routines are stored as a (preferably open-ended) numbered set. The original or any one of the alternatives can be selected for use at runtime (for a list of functions in our RAMP see table 2).
3. RAMP implementations also provide an application programming interface (API) for both remote and on-board scripting. This API enables control of all user aspects of the simulation, including the parameter set, run management, RAM options and data retrieval. Script logic can alter parameter settings and RAM options as the simulation progresses. A Nashorn-based Javascript interpreter enhanced with API methods is provided.
4. The API can be accessed remotely using operating system facilities by external applications running concurrently with the simulator. Of particular, interest is the ability to control the simulation from the R statistical platform. An R routine can be formulated to both manage the simulation run and to retrieve and process the resulting data. The RAMP simulation becomes a ‘virtual package’ to the controlling R logic. See electronic supplementary material, appendix B.

**Table 2.** Parameter values used to simulate single and multivariant outbreaks.

parameter	symbol	value	source/comment
*single-variant simulations*			
time unit	*t*	daily	empirical data are daily
nominal pop size	*N*_0_	10^5^–10^7^	see §3.1^a^
effective contact rate^c^	*κ*_0_	3 per day	implies *R*_0_ ≈ 3.1^b^
transmission	*β*	0.3	implies *R*_0_ ≈ 3.1^b^
latent period	*σ*_*E*_	4 days	median time in E^d^
infectious period	*σ*_*I*_	7 days	median time in I^e^
immunity half-life	*t*^half^	1/2 to 1 year^f^	per run specs.^g^
disease-induced mort.^j^	*p*_*α*_	2% of cases^h^	mortality rate is $\alpha^{i}$
adaptive contact param.	$p_{I}^{\mathrm{half}}$	0, 0.002, 0.05	decreasing *κ*(*I*)^k^
seasonal fluctuation param.	*δ*_season_	0	seasons ignored^l^
***multi-variant simulations*** (single-variant parameter values used where applicable)			
mutation factor^m^	*μ*	0.001^n^	see electronic supplementary material, equation (A.13)
variant number	*j* = 0, …, 2^*J*−1^	*J* is 0 to 7	i.e. 2 to 128 variants
cross-immunity	*c*_*mj*_	0.8	equations (2.1), (2.2)
pathogen shedding	${\bar{\zeta }}_{\;j\ell }$	0.001^n^	see electronic supplementary material, equation (A.9)
environmental persistence	${\bar{\eta }}_{\;j}$	1 for all *j*	see electronic supplementary material, equation (A.10)
transmission	${\bar{\beta }}_{j}$	*δ*_*β*_ ∈ [0, 0.2]	see electronic supplementary material, equation (2.4)
within-host replication rate	*λ*_*j*_	1 for all *j*	see electronic supplementary material, equation (A.13)
disease-induced mort.^o^	$p_{\alpha_{j}}$	0.02	see electronic supplementary material, equation (2.5)

^a^In particular see §3.1.3.

^b^See electronic supplementary material, equation (A.26).

^c^See electronic supplementary material, equation (A.7).

^d^Reciprocal of *γ* in continuous time computation of *R*_0_ per electronic supplementary material, equation (A.26).

^e^Reciprocal of *ρ* in continuous time computation of *R*_0_ per electronic supplementary material, equation (A.26).

^f^Based on statement in [23]: ‘· · · studies of animal coronaviruses antibody titers · · · waned substantially 1 year after initial infection · · · and many could be reinfected and shed virus · · ·’.

^g^See electronic supplementary material, equation (A.6): note *w*(*t*) switches from 1 to 0 as immunity goes from complete to absent.

^h^Value at start of the pandemic, but typically lower later in most regional epidemics.

^i^This is the ‘virulence’ parameter of continuous-time SEIR models.

^j^If *α* ≪ 1 then $p_{\alpha_{j}}=1-e^{-\alpha }\approx \alpha$.

^k^See electronic supplementary material, equation (A.7). Setting $p_{I}^{\mathrm{half}}=0$ implements *κ*(0) = *κ*_0_, though *κ*(*t*) → *κ*_0_ as $p_{I}^{\mathrm{half}}\to \infty$.

^l^Implies values of *k* and *θ* in electronic supplementary material, equation (A.10) are irrelevant.

^m^Variant independent—variant dependence requires more elaborate model.

^n^Quantifies the mutation rate observed at a population rather than within-cell replication level.

^o^If *α*_*j*_ ≪ 1 then $p_{\alpha_{j}}=1-e^{-\alpha_{j}}\approx \alpha_{j}$.

We implemented our RAMP using Java and made ample use of all of the features described above. Use of the RAM facility permitted experimentation with the several versions of cross-immunity (e.g. equations (2.1) and (2.2)). A Javascript program was used to control an adaptive vaccination strategy. A small R package serving as a driver was used in an R program that ran the simulations multiple times, extracted results into R data structures, and produced graphs showing statistical mean and standard deviation. More details on the graphical structure and implementation of our M-SEIR are provided in the presentation of both the results below and information in electronic supplementary material, appendix B.

### 2.3. Simplifications and running the model

In the examples presented in the next section, we have not taken advantage of the full complexity of the model. Thus, for example, in our multivariant simulations, we have assumed that all variants are shed at the same relative rate (i.e. *ζ*_*ij*_ = 1 for relevant *i* and *j* = 0, …, 2^*J*^ − 1), have the same environmental persistence properties (i.e. *η*_ℓ_ = 1 for all ℓ = 0, …, 2^*J*^ − 1), the same within host replication rates (i.e. *λ*_ℓ′_ = 1 for all ℓ′ = 0, …, 2^*J*^ − 1), and are all equally virulent (i.e. *α*_*j*_ = *α* for all *j* = 0, …, 2^*J*^ − 1). Obviously, these assumptions can be relaxed once suitable data are available for a particular pathogen to support variant specific shedding, persistence, within-host replication and virulence values.

If suitable cross-neutralization data are unavailable, then some assumptions must be made regarding the parameter values *c*_*mj*_ in the cross-immunity matrix **C**. For example, we consider the following two contrasting cross-immunity scenarios with respect to a global cross-immunity constant *c* ∈ (0, 1). The first we call *cascading* cross-immunity because the level of cross-immunity diminishes multiplicatively with genetic distance of the two strains: viz.

2.1 $$\begin{array}{ll} & \mathrm{Cascading}\;C\;:\;\\ & \;c_{mj}=\left\{ \begin{array}{ll} 1 & \mathrm{if}\;j=m\;\\ c^{k} & \mathrm{if}\;j\;\mathrm{differs}\;\mathrm{from}\;m\;\mathrm{by}\;k\;\mathrm{alleles}. \end{array}\right. \end{array}$$

The second we call *escaping* cross-immunity because when the final allele in the array of *J* loci mutates from 0 to 1, it escapes completely from cross neutralization effects with all strains that have the original allele at the *J*th locus: viz.

2.2 $$\begin{array}{ll} & \mathrm{Escaping}\;C\;:\;\\ & \;c_{mj}=\left\{ \begin{array}{ll} 1 & \mathrm{if}\;j=m\;\\ 0 & \mathrm{if}\;j\geq 2^{J-1}\;\mathrm{and}\;\ell <2^{J}\;\mathrm{provided}\;J>2\;\\ c^{k} & \mathrm{otherwise},\;\mathrm{where}\;j\;\mathrm{differs}\;\mathrm{from}\;m\;\mathrm{by}\;k\;\mathrm{alleles}. \end{array}\right. \end{array}$$

This is an idealization of the *escape mutation* phenomenon, which we set up here to enable us to evaluate behaviour of such mutations. For the purposes of this paper, idealized escape mutations are defined as those whose level of cross-immunity with the variants from which they arise is 0 (in reality some small level of cross-immunity may remain).

Also, in the absence of comprehensive data that allow us to use realistic estimates for the relative transmissibility *β*_*j*_ and virulence *α*_*j*_ of various variants *j* = 0, …, *J*, we employ the following scenario-facilitating formulations. These permit us to investigate the potential impacts of increased transmission and virulence with the emergence of new strains based on the number of mutations *d*_*j*_ needed to get from variant 0 to variant *j*. Specifically, for

2.3 $$d_{j}=\mathrm{Hamming}\;\mathrm{distance}\;\mathrm{between}\;\mathrm{variants}\;0\;\mathrm{and}\;j,$$

and for transmissibility and virulence perturbation parameters $\delta_{\beta }$ and $\delta_{\alpha }$, respectively, we define

2.4 $$\mathrm{Transmissibility}\;:\;\;{\bar{\beta }}_{\;j}=\beta {\left(1+\delta_{\beta }\right)}^{d_{j}},$$

(the bar notation here reminds us that this value is used in the computation of *β*_*ij*_ according to electronic supplementary material, equation (A.8)) and

2.5 $$\mathrm{Virulence}\;:\;\;p_{\alpha_{\;j}}=p_{\alpha_{\;j}}{\left(1+\delta_{\alpha }\right)}^{d_{j}},$$

(this is a probability rather than a rate and we have to ensure $\delta_{\alpha }$ is selected such that $p_{\alpha_{\;j}}{\left(1+\delta_{\alpha }\right)}^{J}\leq 1$).

Also for simplicity’s sake, we assumed that infectee with major variant *j* will shed minor variants in the immediate neighbourhood of *j* at comparative rate *ζ* ∈ [0, 1) and be major variant-independent: i.e. we assumed

2.6 $$\mathrm{shedding}\;:\;\;\zeta_{\;j\ell }=\left\{ \begin{array}{ll} 1 & \mathrm{if}\;\ell =j\;\\ \zeta & \mathrm{if}\;\ell \;\mathrm{differs}\;\mathrm{from}\;j\;\mathrm{by}\;\mathrm{one}\;\mathrm{allele}\\ 0 & \mathrm{otherwise}\; \end{array}\right.$$

Finally, in this paper, we will not investigate any seasonal effects, which is equivalent to setting *δ*_season_ = 0 in electronic supplementary material, equation (A.10), and using this setting in all our simulation.

The model itself can be accessed at Github (https://github.com/rmsalter/SEIVAgent_distribution), where instructions are available for launching and using our SEIVAgent application.

## 3. Illustrative examples

### 3.1. Single variant simulations

#### 3.1.1. Parameter values and baseline run

The first variable that needs to be determined is the unit of time we use for our simulations because all process rate parameters are scaled by its selection. As the time resolution of empirical COVID-19 incidence and mortality data is daily, we selected our unit of time *t* to be days. Additionally, based on various sources including a metapopulation study of COVID-19 parameter estimates [39], we set the latent and infectious periods to be 4 and 7 days, respectively. Basic SEIR epidemiological models do not separate out the processes of contact and transmission-per-contact, so we had some leeway on what values to choose for contact rates and transmission rates per contact because it is the value of the product of these that is important in determining the reproductive value, commonly referred to as ‘*R*_0_’ for COVID-19. Hussein *et al.*’s [39] meta analysis of COVID-19 zeroed in on *R*_0_ = 3.14 as a mean value across a range of studies (95% confidence interval [2.69, 3.59]). By setting our baseline contact rate and transmission parameters to be *κ*_0_ = 3 and *β* = 0.3, we estimated the value of *R*_0_ in our model to be approximately *R*_0_ = 3.1. These and the remaining values of the parameters used in our simulations are summarized in table 2.

#### 3.1.2. Adaptive contact rate

None of the major outbreaks of COVID-19 around the world followed a classic ‘rise-and-fall’ incidence curve because of social distancing and other measures taken to mitigate transmission once it had been determined that a full-blown outbreak was underway. These measures waxed and waned with government regulations and the perception that the outbreak was respectively under or out of control. This, in turn, resulted in incidence profiles that rose and fell multiple times (i.e. so-called waves) as these measures waxed and waned. Thus, it is not possible to replicate the incidence curves of any of the country/regional epidemics without characterizing the social distance and subsequent social relaxation measures driving their rise and fall [15].

In a general way, we can capture the dominant feature of this kind of social behaviour by assuming the contact rate *κ*(*t*) is influenced by current or recent prevalence levels, where current prevalence is given by the ratio of the number of infected individuals *N*_*I*_(*t*) to current population size *N*(*t*) − *N*_*D*_(*t*). Thus, in the various scenarios present below, we assume an adaptive response rate that has a maximum value *κ*_0_ when *I*(*t*) = 0 and is reduced to half this value, as a declining sigmoidal curve specified in electronic supplementary material, equation (A.7), when $N_{I}(t)/\left(N(t)-N_{D}(t)\right)=p_{I}^{\mathrm{half}}$. If we simulate the first year of an epidemic using our basic parameters (table 2; also see parameter panel in figure 2) and adaptive contact half-max parameters for the cases $p_{I}^{\mathrm{half}}=0.01$ and 0.02 (i.e. 1% and 2% prevalence, respectively), we obtain the per cent of susceptibles (uninfected) and cumulative deaths by day 365 provided in table 3.

**Table 3.** Basic runs with one million individuals (*N* = 1 000 000) using two different half-max adaptive contact parameter values $p_{I}^{\mathrm{half}}$ compared with listed countries.^a^

	$p_{I}^{\mathrm{half}}$		USA	Italy	Czechia
	1%	2%	(values under reported)^c^		
uninfected at day 365^b^	82%	71%	93%	95%	88%
COVID-19 deaths by day 365^b^	0.34%	0.55%	0.12%	0.17%	0.21%

^a^Data from Worldometer.

^b^One year after the first 10 recorded cases in the countries concerned.

^c^Substantial under reporting occurs for both cases [40] and deaths [41].

For purposes of comparison, we also provide in table 3 the per cent of susceptible individuals and per cent of deaths due to COVID-19 1 year after the day on which more than 10 cases of COVID-19 were recorded to occur in the USA, Italy and Czech Republic (extracted from data provided at Worldometer (https://www.worldometers.info/coronavirus/country/us/)). As these data are known to be substantially under reported for both cumulative prevalence [40] and deaths [41], we felt that $p_{I}^{\mathrm{half}}=0.01$ (i.e. 1% prevalence) provides a reasonable ballpark value for an adaptive contact rate half-max parameter for our various illustrations provided below.

Finally, it is worth noting that the adaptive contact rate may be much more complicated than we have represented here. For example, social distancing fatigue may set in over time, effectively inducing a time-varying value on the parameter $p_{I}^{\mathrm{half}}$ itself. Our RAMP design allows modellers to readily implement a more complex adaptive response should they choose to do so.

#### 3.1.3. Population size and demographic stochasticity

Deterministic SIR/SEIR and related models will always produce an epidemic whenever the parameters ensure that *R*_0_ > 1 [2,34]. As these models do not include the demographic stochastic effects associated with finite populations, they are unable to capture the phenomenon of stochastic extinction of the epidemic before it gets going when a single infected individual is introduced into an otherwise infected population (with regard to the pathogen in question; see discussion in electronic supplementary material, A.4). In such models, results are either cited using percentages or numbers per thousand or per hundred thousand individuals and the actual population size is not regarded as a factor. Population size, however, is a factor in determining the absolute size of an epidemic once it gets started and deterministic models provided a robust assessment of the course of the epidemic in populations consisting of millions of individuals (other factors, such as spatial or contact network structure play a more important role than size per se [5–7]).

**Figure 3.**
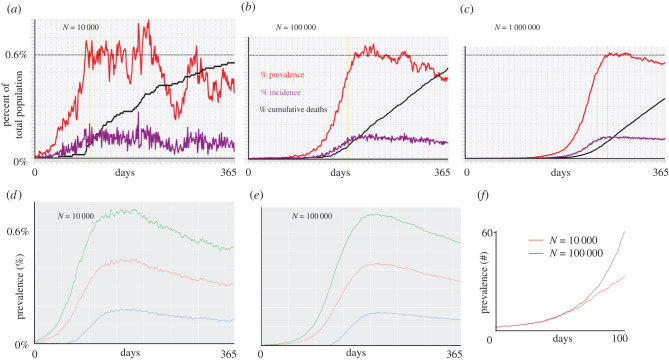
(*a*–*c*) Plots of percentage prevalence (red), incidence (purple) and cumulative dead (black) for 365-day simulations using the parameter values given in table 1 with the adaptive contact rate parameter $p_{I}^{\mathrm{half}}=0.01$ (see electronic supplementary material, equation (A.7) in SOI) and *N* = 10 000, *N* = 100 000 and *N* = 1 000 000, respectively. (*d*–*e*) Plots of mean percentage prevalence (red) over the first year plus (green) minus (blue) 1 s.d. over 100 runs (runtime seeds going from 0 to 99) for the cases *N* = 10 000 and *N* = 100 000, respectively. (*f*) Plots of the actual prevalence (number of individuals) for the first 100 days for the cases *N* = 10 000 (red) and *N* = 100 000 (black).

To get a sense of the effects of demographic stochasticity on populations of different sizes in our simulations, we compared the prevalence, incidence and cumulative deaths obtained for single runs (runtime seed = 1) of a basic adaptive contact rate scenario (basic parameters plus $p_{I}^{\mathrm{half}}=0.01$) for cases where the initial population sizes where *N*_0_ = 10 000, 100 000 and 1 000 000 (figure 3*a*–*c*). We also compared the mean prevalence plus/minus 1 s.d. for 100 runs (runtime seeds ranging from 0 to 99) for the two cases *N*_0_ = 10 000 and 100 000 over both the 1st year (figure 3*d*,*e*) and the first 100 days (figure 3*f*).

The results depicted in figure 3 can be encapsulated in the following four well-established principles:

1. The initial phase of an outbreak is independent of population size and establishment of an epidemic depends solely on the value of *R*_0_ (electronic supplementary material, appendix A.4). Thus, as we see in figure 3*f*, the first 50 days of the mean prevalence for the simulations of the cases *N* = 10 000 and *N* = 100 000 are virtually identical, only departing from one another from around day 50 onwards.
2. Beyond the initial phase, stochastic fluctuations are more evident in smaller than larger populations (compare figure 3*a*–*c*).
3. Ultimate prevalence levels, aside from stochastic fluctuations, are independent of population size. Thus, for example, we see that prevalence maxes out at round 0.6% in all three cases (dotted lines) across a range of two orders of magnitude in population size.
4. Mean population prevalence will always max out at lower levels than the prevalence reached in actual runs (viz. the maximum exceeds 0.7% individual runs in figure 3*a*–*c,* while it is between 0.4% and 0.5% for the red curves in figure 3*d*,*e*) because the mean values take into account the fact a proportion 1/*R*_0_ of the runs go extinct within the first several weeks [42].

### 3.2. Multivariant simulations

**Figure 4.**
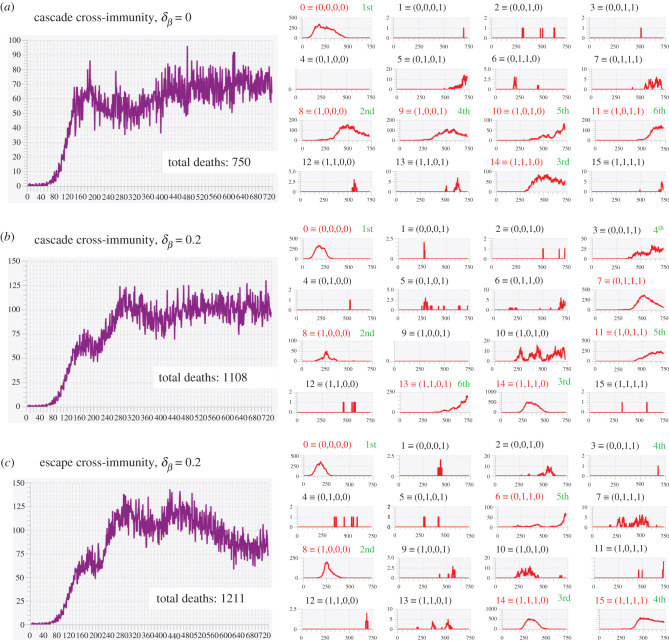
Total daily incidence (Δ*I* +: purple) and variant-specific prevalence (*I*: red) for a 16-variant epidemic in a population of size *N* = 50 000 (for other parameter values see table 2) are plotted over a 2-year period for the three cases: (*a*) cascade cross-immunity $\delta_{\beta }=0.0$ (i.e. all *β*_*j*_ = 0.3, *j* = 0, …, 15), (*b*) cascade cross-immunity $\delta_{\beta }=0.2$ and (*c*) escape cross-immunity (in the two latter cases *β*_0_ = 0.3, *β*_15_ = 0.622 and *β*_*j*_, *j* = 1, …, 14, determined using equation (2.4)). Variant number and corresponding binary representation as labelled in red for dominant or co-dominant variants (incidence at some point greater than 50 individuals per day) and grey for minor variants (incidence always less than 50 over the 2-year simulation). The order of emergence of dominant or co-dominant variants is labelled in green. Note that each panel has its own vertical scale but all plots are over 730 days (even in cases where the horizontal axis label go to 750).

We carried out a series of multivariant simulations with *J* = 4 (i.e. 16 variants can emerge) in a population of size *N* = 50 000. We compared the scenarios of cascading cross-immunity with *c* = 0.8 (equation 2.1) and transmission rates the same for all variants (figure 4*a*) with the same cascading cross-immunity as in figure 4*a*, but now we allowed transmission to increase by 20% for each mutation difference between variant *j* and variant 0 (equation (2.1); *β*_*j*_ = 0.3, *j* = 0, …, 15, $\delta_{\beta }=0.2$ and *β*_*j*_, *j* = 1, …, 15, determined using equation (2.4)). Finally, we compared the scenario of cascading cross-immunity with that of escaping cross-immunity for the case *c* = 0.8 (equation 2.2) and obtained the results provide in figure 4*c*. The severity of each scenario is encapsulated in the total death statistic over the course of the 2-year simulation.

Our primary observations comparing the results plotted in figure 4*a*–*c* and other runs (not shown here) of the same scenarios using different runtime seeds, are the following:

— In all three cases the initial variant, by construction, is 0 ≡ (0, 0, 0, 0). In our three scenarios, this variant was followed by chance by the emergence of variant 8 ≡ (1, 0, 0, 0), but this is common to all three scenarios because they use the same sequence of pseudo random numbers in their simulations. Using different runtime seeds, however, leads variants other than variant 8 emerging to replace variant 0. Thus, the mutant identity (i.e. its binary representation) of the variant to first emerge is somewhat random, but it is going to be influenced by having different transmission values for each variant (scenarios (*b*) and (*c*)), as well as the possibility of an idealized escape mutation (scenario (*c*)).
— We expect variants that have an idealized escape mutation to emerge early, as is the case in scenario (c) in which variants 8–15 have the idealized escape mutation. In particular, in figure 4*c*, we see that the second to fourth variants to emerge all have the idealized escape mutation (i.e. variant 8 then 14 and then 15), and finally variant 6 ≡ (0, 1, 1, 0) emerges because of the cross-immunity between all variants with the idealized escape mutation finally comes into play.
— When *δ* > 0, the variants with the higher values of *β* come to dominate, though they take time to emerge. In our cascading cross-immunity case with $\delta_{\beta }=0.2$, the most transmissible of these (variant 15 ≡ (1, 1, 1, 1)) had yet to emerge within the simulated 2-year period. The existence of the idealized escape mutation, however, does facilitate the emergence of variant 15 at the beginning of the second year (figure 4*c*). Another run of this scenario (runtime seed = 1; results not shown here) has variant 15 emerging very early (around day 120). Furthermore, due to the effects of cross-immunity, this variant was replaced by variant 11 ≡ (1, 0, 1, 1) around days 450–500. Variant 15, however, as result of waning and cross-immunity effects, reemerges again around day 600, with variant 11 declining over the last three months of the second year.

### 3.3. Vaccination rollout

#### 3.3.1. Single valency vaccinations

As illustrations of potential issues associated with the design and implementation of vaccination programs, we first considered vaccinating individuals in a population of 100 000 subject to an epidemic involving a single variant of the pathogen. We note that in a population of *N* = 100 000 individuals, a vaccination rate of *v*(*t*) = 0.001 involves vaccinating an average of 100 individuals per day with daily variation following a binomial distribution (i.e. a standard deviation of just under 10 individuals per day).

**Figure 5.**
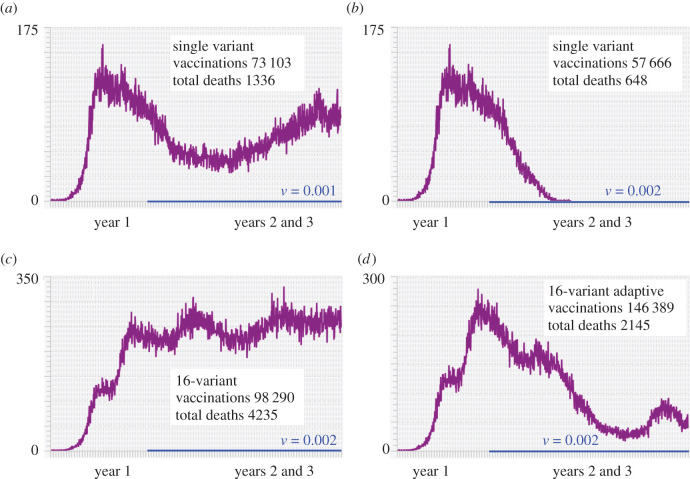
Incidence (Δ*I*^+^: purple) is plotted over 3 years for the baseline run (parameters given in table 1 with *N* = 100 000) for the cases where vaccination rates *v*(*t*) (indicated by blue lines) are applied during the second and third years only to individuals not previously vaccinated but otherwise selected at random (for clarification, the average number of individuals vaccinated each day is 100*v*(*t*)% with variation following a binomial distribution). Our first two simulations involve vaccination rollout programs in a single variant epidemic at vaccination rates (*a*) *v*(*t*) = 0.001 and (*b*) *v*(*t*) = 0.002 (respectively, 0.1% and 0.2%) of individuals not previously vaccinated, but otherwise chosen at random. Our second two simulations involve vaccination rollout programs in a 16-variant epidemic, both at vaccination rates *v*(*t*) = 0.002, involving (*c*) individuals not previously vaccinated and (*d*) a bivalent adaptive vaccination program in which previously vaccinated individuals could be vaccinated again with a new valency vaccine, as described in the text.

Rollout of our vaccination program began on day 366 after the start of the outbreak and ran for 2 years beyond that to day 1100 (figure 5). Such scenarios place us within the context of the COVID-19 epidemic because vaccinations were only available from around the second year onwards. In our first two scenarios, we selected individuals, respectively, at rates 0.1% (*v* = 0.001) and 0.2% (*v* = 0.002) of the population each day (figure 5*a*,*b*). Only individuals who had not been previously vaccinated were selected, but selection was otherwise random.

Additionally, we simulated a 16-variant scenario in which individuals were vaccinated at against variant 0 at rate *v* = 0.002 (figure 5*c*). Again, individuals were selected at random from the set of those who had not been previously vaccinated. By vaccinating individuals against variant 0, some immunity was conferred against variants 1–7 through cross-immunity relationships according to the Escaping **C** case with cross-immunity parameter *c* = 0.8 (equation 2.2). In this scenario, variants 8–15 contain the idealized escape mutation.

Our focal question with regard to the first two scenarios was: What vaccination level is needed to extinguish the epidemic in the population encompassed by the vaccination rollouts for the populations concerned? From these two simulations (figure 5*a*,*b*), we see that vaccination rate *v*(*t*) = 0.001 was insufficient to eliminate the pathogen from the population, while *v*(*t*) = 0.002 was able to eliminate the pathogen within 10 months from the start of the vaccination rollout. Furthermore, in the first of these simulations (figure 5*a*), we see a resurgence of incidence in year three, which implies that the effects of waning immunity in this case are essentially ‘outrunning the vaccination rate.’

Our focal question with regard to a comparison of scenarios two and three (figure 5*b*,*c*) was: does the vaccination rate *v*(*t*) = 0.002, which was able to exterminate the outbreak in the 1-variant case, remain able to exterminate the outbreak in the 16-variant case when an idealized escape mutation is involved? The answer to this question from the observed incidence curve (figure 5*c*) is a resounding no. In fact, the total death rate over the 3-year period rose from 0.13% of the population (1336 individuals) to 0.42% of the population (4235 individuals).

#### 3.3.2. Adaptive bivalent vaccinations

With the emergence of new variants, the possibility exists to modify vaccines to contain or induce the production of antigens that directly target the variant in question (i.e. rather than through cross-immunity that arises when a related variant is the direct target) [28]. Furthermore, it is possible for vaccines to be multivalent in terms of directly targeting more than one variant at time [28]. In our third vaccination scenario, a univalent vaccine applied at a rate *v* = 0.002 failed to bring a multivariant epidemic under control. Thus we were motivated to explore a scenario to see what would happen with a bivalent vaccine that was implemented adaptively in the sense of its two valencies following the two dominant variants.

In the specific vaccination rollout program that we employed in our fourth simulation, we did not account for logistical, production and variant monitoring constraints. Such constraints, of course, exist and vary across locations and populations: in real applications, they need to be taken into account. The program we employed assumes that we are able to alter the valency of the vaccination used every 15 days, based on an ability to identify the two variants that are most prevalent at each of these successive 15-day-apart observation points (from day 365 to day 1085, which is the start of the last 15-day period ending just prior to the start of day 1100). If only one variant had an incidence exceeding 9 individuals on an observation day, then the vaccination applied over the next 15-day interval was monovalent for the dominant variant, otherwise it was bivalent for the two variants that had the highest incidence on that observation day.

As with the non-adaptive vaccination rollouts, individuals were selected at random from a pool that had previously not been vaccinated with the particular valency-specific vaccine (either bivalent or monovalent). However, in the bivalent vaccine case, if an individual had previously been vaccinated to only one of the two variants defining the latest vaccine, then these individuals were incorporated into the vaccination pool from which individuals were randomly selected for vaccination. If such individuals were selected then the start of the waning time relating to the previous vaccination was reset to start anew. Thus, with this program, it is possible for individuals to be vaccinated more than once.

The results of this simulation are depicted in figure 5*d*, where we see that this vaccination program is much more effective in preventing deaths than the monovalent variant 0 program applied to the same 16-variant epidemic at the same vaccination rate in figure 5*c* (total deaths are 4235 in the former versus 2145 in the latter case). The valencies of the vaccine applied during each new 15-day period are listed in table 4. We note that the monovalent case involves considerably fewer vaccinations because of the ‘no revaccination with the same vaccine’ restriction in our rollout program. In particular, over 2 years of vaccinating at a 0.2% rate per day, all individuals are vaccinated in the case of figure 5*c* by day 859, while in the case of figure 5*d* revaccinations kept occurring as individuals who have not previously been vaccinated to one of the focal variants were revaccinated. Even in this adaptive rollout, however, the epidemic was only substantially lowered rather than completely extinguished. The latter event for the set of parameters used in our simulation requires a somewhat higher vaccination rate than 0.2% per day; or, perhaps it requires lower rates of waning immunity, higher cross-immunity rates, or the lack of an idealized escape mutation. All of these effects can be demonstrated through the selection of appropriate parameter values, but the specifics are only relevant when the model is applied in a real-world situation.

**Table 4.** Valency of adaptive vaccination over the interval 365 to 1100 days.

time (days)	valency
(365, 470)	(9, 14)
(470, 530)	(13, 14)
(530, 680)	(13, 15)
(680, 740)	(15)
(740, 905)	(10, 15)
(905, 1025)	(10, 12)
(1025, 1070)	(12, 15)
(1070, 1100)	(15)

## 4. Discussion

The amount of structure and data needed in complex biological systems’ models depends on the questions that these models have been formulated to address [13,14]. In this paper, we steered away from making specific predictions—because universal solutions are not always locally applicable. Rather, we focused on gaining insights into incidence patterns that can be expected when contacts are adaptive rather than fixed, multiple variants may emerge (typically sequentially over time), and open versus adaptive uni- and multivalent vaccination programs are implemented to try to eliminate local pandemics. Analyses that incorporate more complexity in the hopes of attaining greater realism, such as adding heterogeneity related to age and spatial structures, as well as behavioural and social groups, require data that are specific to a local population (town, city, county or small country, etc.). Such elaborations are only worth incorporating when the study relates to a real world system supported by adequate data (the latter related to the complexity of the question that needs to be addressed, as discussed elsewhere [13]).

In the future, we hope to release versions of our RAMP that include both demographic (e.g. age, job, or state-of-health categories) and spatial structures (e.g.using metapopulations/network formulations). We stress, however, that the number of metapopulation nodes and demographic categories leads to a quadratic proliferation of parameters because transmission modifications by demographic-category pairs and metapopulation-nodal pairs need to be included through the elements of mixing matrices [7,43].

Beyond these elaborations, structure can be added to address other salient issues. One such issue would be to obtain a better understanding of the role informational delays may play in producing the type of incidence waves that have been observed over the course of the COVID-19 pandemic (and as we have modelled in [44]). Such delays would lead to contact rates containing a time-lag rather than depending only on current prevalence levels. We might also spend more time deconstructing the relative importance of such time delays versus the emergence of more transmissible variants in accounting for these waves.

Beyond gaining a deeper understanding of some of the mechanisms responsible for the incidence patterns observed among local epidemics of the COVID-19 pandemic, a second and primary purpose of our paper is to present our M-SEIR RAMP as a platform that others may use to address issues of concern to them in formulating policies to manage local COVID-19 epidemics. This also has the advantage of providing an exemplar of our novel RAMP concept and the methods we used to construct it. At this time, the primary value of our M-SEIR RAMP itself may be in testing various vaccination strategies as they relate to variant emergence [45]. Clearly, such applications would require more specific variant-related information on the comparative transmissibility *β*_*j*_, virulence *α*_*j*_, shedding (${\bar{\zeta }}_{\;jm}$), environmental persistence (${\bar{\eta }}_{j}$) and within- host replication rates (*λ*_*j*_) of newly emerging variants.

Equally important, though, in evaluating the impacts of vaccination strategies on local epidemics is obtaining variant-specific immunity and cross-immunity data. This includes waning rates, which we have not made variant specific. Our model, however, could be generalized to include variant-specific waning rates represented by the parameter $t_{j}^{\mathrm{half}}$ (electronic supplementary material, equation (A.6)). It also includes information for characterization of the elements *c*_*j*ℓ_ of the cross-immunity matrix **C** (i.e. a generalization that renders equation (2.1) redundant). Models are sorely needed to explore multivariant dynamics, particularly the epidemiological properties regarding shedding, environmental persistence, transmission, mutation and within-host replication rates. These processes, acting together, determine the relative success of different variants and their actual impact on the severity of epidemics and the nature of vaccination programs needed to suppress them.

Making our model both location- and variant-specific could be undertaken using methods, such as appropriate complexity modelling [13,14], designed to enhance the relevance of models. Furthermore, in some cases, it may be useful to add spatial or age-structure information to our M-SEIR or include a contact network [7], which itself may contain spatial or refined class category (e.g. age or work categories) information. In addition, our current implementation represents variant differences in terms of *J* loci with two alleles (denoted by 0 and 1, respectively) at each locus. A more realistic representation of the genetic basis of variant differences may involve genetic representations in which several alleles are possible at each locus. Furthermore, the loci themselves may represent relevant molecular structures such as epitopes.

An advantage of our RAMP design features is that they provide a framework for elaborating or simplifying model details in the pursuit of different questions at various points in a pandemic. For example, suppose we are interested in pursuing inferences regarding the drivers of variant evolution at various stages of the pandemic. We may first want to address questions relating to pandemic behaviour, driven by mutations that increase transmissibility. This is what actually happened with the appearance of the SARS‐CoV‐2 D614G and the alpha variants. A year or so into the pandemic, however, we may then want to explore processes that give rise to immunity-escaping variants. This, again, is what happened in reality. Our RAMP formulation gives us the flexibility to change the model part way through a pandemic. In particular, we can then test which among a set of alternative reinfection processes is most likely to produce an escape mutation once reinfection begins to occur on a substantial scale. By configuring model drivers so that we first have a relatively simple evolutionary process and then switch to more complex evolutionary processes, our RAMP design facilitates comparing several competing explanations of observed patterns of variant emergence at different stages of a pandemic.

Although cross-immunity and immune waning are entangled in our immunity modifier functions (i.e. *ϕ*_*ij*_; see electronic supplementary material, equation (A.8)), cross-neutralization data can be used to estimate the cross and waning immunity parameters using appropriate methods [46]. Such data are becoming more widely available through the application of improved serological and genetic methods [24,47,48]. Variant and cross neutralizing studies bring up a much neglected issue, which is the effect of dose (number of pathogens involved in the initial infection, also known as viral load) on the severity of the infection. Furthermore, dose affects both the probability of host invasion (in the context of transmission), as well as mutational rates once host invasion has occurred. Effective dose differs from the questions of the number of vaccine doses (typically one versus two) versus the antigen or virus-like particle load in each dose [49]. In the context of vaccination, both these issues and the technology used to produce the vaccine [28] may well have an impact on waning immunity rates and cross-immunity values. Thus, the parameter values used in the model should ultimately be vaccine-specific, once vaccine-specific waning data have been obtained.

In the coming years, as we obtain more information on the nature of waning and cross-immunity to different variants of SARS-CoV-2, not to mention the vaccines as well, it will become more apparent to us whether or not COVID-19 will settle into global endemicity [32,50] and require periodic vaccinations to combat new variants, as they arise over time. If this is the case, then constant vigilance and a well-designed vaccination program with respect to vaccinating the young and implementing booster vaccinations with appropriate variant-valency will become the order of the day. Additionally, we anticipate extending our M-SEIR RAMP to include RAMs designed to compute the optimal time to administer vaccine booster shots of the same or different variant valencies. Implementation of these RAMs can play a decisive role in the rational design of effective and efficient COVID-19 vaccination programs worldwide. The need for efficacy is made apparent from the fact that our simulations suggest that it may be harder than currently anticipated to eliminate COVID-19 using non-adaptive vaccination programs.

Finally, our M-SEIR RAMP, with its RAMs, driver scripts and ability to be integrated with R and other software platforms and a JavaScript simulation driver window, provides the first example of a new concept in model implementation that facilitates model sharing and easy modification by users other than the original developers. We believe such platforms can come to play an important role not only in disease modelling, but in all fields of research that rely on models for comprehensive analyses of the behaviour of systems of interest.

## Data accessibility

This article has no additional data.
